# ctDNA Detected after Neoadjuvant Therapy for HER2-Positive Breast Cancer Is Associated with Inferior Outcomes and May Inform Adjuvant Therapy

**DOI:** 10.1158/2767-9764.CRC-24-0234

**Published:** 2026-01-14

**Authors:** Po-Han Lin, Li-Wei Tsai, Chiao Lo, Sung-Hsin Kuo, Chia-Chun Ni, Chih-Hao Yu, Chiun-Sheng Huang

**Affiliations:** 1 Department of Medical Genetics, National Taiwan University Hospital, Taipei, Taiwan.; 2 Institute of Medical Genomics and Proteomics, College of Medicine, National Taiwan University, Taipei, Taiwan.; 3 Department of Surgery, National Taiwan University Hospital and Medical College of the National Taiwan University, Taipei, Taiwan.; 4 Department of Oncology, National Taiwan University Hospital and Medical College of the National Taiwan University, Taipei, Taiwan.

## Abstract

ctDNA has been reported as a predictor of the progression of metastatic breast cancer and recurrence in early breast cancer (EBC). This study explored whether ctDNA persistence in patients with HER2-positive (HER2+) EBC who achieved pathologic complete response (pCR) after neoadjuvant therapy (NAT) indicates a poor prognosis and the need for adjuvant T-DM1. Of the 117 patients enrolled, 25 patients achieved pCR after NAT and 92 patients did not. Six of the 25 pCR patients showed positive ctDNA after NAT, whereas 26 of the 92 non-pCR patients were ctDNA-positive. Eighteen patients received adjuvant T-DM1, whereas 99 patients did not. Among the non–T-DM1 group, ctDNA positivity after NAT independently predicted recurrence (HR, 5.505; 95% confidence interval, 1.950–15.540; *P* = 0.001). pCR patients with ctDNA positivity experienced a shorter recurrence-free survival (RFS) compared with pCR and ctDNA-negative patients after NAT(*P* = 0.008), whereas non-pCR patients with ctDNA-negative tumors had a better RFS compared with non-pCR patients with ctDNA-positive status (*P* = 0.001). In the 79 patients who were ctDNA-positive before NAT, clearance of ctDNA by NAT was associated with significantly better RFS than nonclearance (*P* < 0.001). Adjuvant T-DM1 also significantly improved the ctDNA clearance rate (*P* = 0.035) compared with non–T-DM1 therapy in patients with ctDNA positivity after NAT during serial tests. We classified the 117 patients as T-DM1/non–T-DM1 and ctDNA-positive/negative, and a significantly shorter RFS was observed in patients with ctDNA-positive/non–T-DM1 (*P* = 0.029) than in the other three patient groups. In conclusion, the presence of ctDNA after NAT in patients with HER2+ EBC is associated with a poor prognosis and may indicate adjuvant T-DM1.

**Significance:**

Adjuvant T-DM1 can improve the survival of HER2+ EBC patients who don’t achieve pCR. Our study showed that ctDNA positivity after NAT was associated with decreased RFS. ctDNA-positive status after NAT can be switched to ctDNA-negative status in patients treated with T-DM1. Patients who were ctDNA-positive and treated with T-DM1 had a similar RFS to those who were ctDNA-negative, indicating that “ctDNA positivity” could be a marker determining adjuvant T-DM1 therapy.

## Introduction

Patients with human growth factor receptor 2–positive early breast cancer [HER2-positive (HER2+) EBC] who achieve pathologic complete response (pCR) after neoadjuvant therapy (NAT) have a lower recurrence rate than those without pCR (1–3). Adjuvant T-DM1, an antibody–drug conjugate (4), has been shown to improve survival in patients who do not achieve pCR after NAT, compared with trastuzumab (5). Consequently, NAT has become the standard of care for most HER2+ EBC cases, and pCR is a key predictor of recurrence and an indication for adjuvant T-DM1 (6). However, despite achieving pCR, some patients experience recurrence or distant metastasis (7–9), whereas a proportion of patients without pCR maintain long-term disease-free status (10). This observation highlights the need for a precise biomarker to predict recurrence (11).

ctDNA is a biomarker that can be used to detect cancer recurrence early, predict disease progression, and monitor resistance (12, 13). A prognostic role was observed in the assessment of perioperative ctDNA levels in patients with colon cancer who underwent hepatectomy for liver metastasis and in those with EBC treated with NAT (14, 15). The I-SPY2 trial and our previous study identified the presence of ctDNA after NAT as an independent factor associated with breast cancer recurrence (10, 16). We previously observed two patients who achieved pCR but harbored ctDNA after NAT and subsequently developed distant metastases. Conversely, patients without pCR but with ctDNA clearance after NAT showed favorable outcomes (10, 16). This disparity suggests that whereas pCR assessment focuses solely on the primary breast tumor without evaluating systemic micrometastatic tumor cells and may miss residual disease, ctDNA analysis provides a comprehensive evaluation of the disease status (17). In other malignancies, ctDNA-negative results have been associated with a low recurrence risk in patients with stage II colon cancer, rendering adjuvant chemotherapy unnecessary (18). Additionally, ctDNA-positive results after treatment have proven effective in predicting early recurrence in patients with non–small cell lung and colon cancers (19–21). Thus, ctDNA could be a robust marker for predicting recurrence or metastasis (11–13, 16, 20–22).

Despite the potential of ctDNA as a biomarker for cancer recurrence, its role in selecting adjuvant regimens after NAT for HER2+ EBC remains unclear. To the best of our knowledge, this is the first study to explore the prognostic role of ctDNA and its utility in guiding adjuvant therapy for HER2+ EBC.

## Materials and Methods

### Patients and samples

We conducted an ancillary ctDNA study on patients participating in a prospective observational study titled, “Development of molecular pathology model and recurrence prediction tool for the Taiwanese breast cancer population.” This study was conducted in accordance with the Declaration of Helsinki and its later amendments or comparable ethical standards. This study was approved by the Medical Ethics Committee of the National Taiwan University Hospital (201809075RSD), and all patients had written the informed consent.

All patients included in this analysis were histologically confirmed to have HER2+ breast cancer, with either 3+ HER2 staining or a score of 2+, as confirmed by fluorescence *in situ* hybridization. The clinical stage at initial diagnosis was confirmed using whole-body CT, and patients with distal metastatic disease were excluded.

A total of 117 female patients with early stage HER2+ breast cancer who received NAT between January 1, 2014, and December 31, 2021, were included. The NAT was a chemotherapy and anti-Her2 antibody-based regimen, including at least four cycles of taxane chemotherapy and trastuzumab or dual Her2 blockade (trastuzumab plus pertuzumab). The end of the follow-up period was Aug 2022. Data analysis was performed between September 1, 2022, and October 30, 2022. Perioperative ctDNA collection was performed as previously described (15). Briefly, we collected 10 mL of blood from each patient at initial diagnosis before NAT (referred to as “before NAT”). Another 10 mL of blood was sampled after NAT and breast surgery (referred to as “or after NAT”). Cell-free DNA was extracted using the MagMAX Cell-Free DNA Isolation Kit (Thermo Fisher Scientific) according to the manufacturer’s protocol.

The pathologic responses were evaluated by specific breast pathologists. pCR was defined as the total clearance of invasive carcinoma in the primary breast tumor and axillary lymph nodes (5). Following surgery, the patients received adjuvant anti-HER2 targeted therapy, including trastuzumab alone, dual blockade (trastuzumab plus pertuzumab), or T-DM1, based on pCR, treatment affordability, trial availability, and finally the decision of the patients. The overall duration of anti-HER2 targeted therapy was 1 year, based on the guidelines of the National Comprehensive Cancer Network Breast Cancer. The decision about the necessity of local radiotherapy depended on factors such as the type of surgery and number of positive lymph nodes. Adjuvant hormone therapy was administered to patients positive for estrogen and/or progesterone receptors.

All patients underwent regular follow-up at the breast clinics, including annual mammography, breast ultrasound, chest radiography, and semiannual blood biochemistry examination. Whole-body image or bone scan was performed when the presence of symptoms was observed. Serial blood samples for ctDNA were collected from some patients every 6 to 12 months during scheduled visits.

### Sequencing and bioinformatics of ctDNA

Barcoded libraries were prepared from 10 ng of cell-free DNA using Ion Torrent Oncomine Breast cfDNA Research Assay v2 (Thermo Fisher Scientific). This panel includes the hotspot regions of *AKT1*, *CCND1*, *EGFR*, *ERBB2*, *ERBB3*, *FGFR1*, *ESR1*, *FBXW7*, *KRAS*, *PIK3CA*, *SF3B1*, and *TP53*. Emulsion PCR and chip loading were performed using the Ion Chef System, and sequencing was performed on the Ion GeneStudio S5 Prime Instrument (RRID: SCR_017984) using the Ion 540 Kit-Chef system (Thermo Fisher Scientific).

The BAM files aligned to the hg19 human reference genome were generated using a sequencing machine. The bioinformatics pipeline has been described previously (10). GATK was used for variant calling with Mutect2 and various filtration parameters. Finally, ANNOVAR was used to annotate the genetic variants. All variants were confirmed using the Integrative Genomics Viewer.

### Statistics

The *χ*^2^ test and Fisher exact test were used to assess the significance of the variance between each group. Kaplan–Meier analysis was used to estimate recurrence-free survival (RFS). Cox proportional hazards regression analysis was conducted to determine the HRs of RFS with corresponding 95% confidence intervals (CI) for various factors. All *P* values were two-sided, and those less than 0.05 were regarded as statistically significant.

## Results

### Patient characteristics

We enrolled 117 patients with early-stage HER2+ breast cancer who underwent NAT. Of the 117 patients enrolled, 25 (21.4%) achieved pCR after NAT, and 92 (78.6%) did not achieve pCR (Supplementary Fig. S1A). Six (24%) of the 25 patients who achieved pCR showed positive ctDNA after NAT, whereas 26 (28%) of the 92 non-pCR patients were ctDNA-positive. Overall, 79 (67.5%) of the 117 patients tested positive for ctDNA before NAT, and 32 (40.5%) of the 79 patients remained ctDNA-positive after NAT. Thirty-eight of the 117 patients (32.5%) were ctDNA-negative before NAT, and none became ctDNA-positive after NAT (Supplementary Fig. S1B).

Regarding anti-HER2 adjuvant regimens, 18 (15.4%) patients received adjuvant T-DM1, and 99 (84.6%) received non–T-DM1 (70 received trastuzumab alone and 29 received trastuzumab plus pertuzumab). None of the 18 patients who received adjuvant T-DM1 achieved pCR after NAT. Ten of the 18 patients were ctDNA-positive and eight were ctDNA-negative after NAT. Seventy-four of the 99 patients who did not receive adjuvant T-DM1 did not achieve a pCR. Detailed characteristics are listed in Table 1.

**Table 1.** Patient and tumor characteristics according to the postsurgery anti-Her2 therapy.

Characteristics	Overall	Non–T-DM1	T-DM1	*P* value
Overall	117 (100.0%)	99 (84.6%)	18 (15.4%)	​
Age (mean, range)	​	51.4 (9.6)	48.3 (10.8)	0.214
Estrogen receptor	​	​	​	0.478
Positive	56 (47.9%)	46 (39.3%)	10 (8.6%)	​
Negative	61 (52.1%)	53 (45.3%)	8 (6.8%)	​
Tumor size before NAT	​	​	​	0.871
T1	10 (8.5%)	9 (7.7%)	1 (0.8%)	​
T2	80 (68.4%)	67 (57.3%)	13 (11.1%)	​
T3–4	27 (23.1%)	23 (19.7%)	4 (3.4%)	​
Lymph node status before NAT	​	​	​	0.386
Node-negative	29 (24.8%)	26 (22.2%)	3 (2.6%)	​
Node-positive	88 (75.2%)	73 (62.4%)	15 (12.8%)	​
Residual tumor size after NAT	​	​	​	0.063
No tumor/Tis	30 (25.6%)	29 (24.8%)	1 (0.8%)	​
ypT1	55 (47.0%)	42 (35.9%)	13 (11.1%)	​
ypT2	26 (22.2%)	22 (18.8%)	4 (3.4%)	​
ypT3–4	6 (5.1%)	6 (5.1%)	0	​
Lymph node status after NAT	​	​	​	0.341
ypN0	75 (64.1%)	66 (56.4%)	9 (7.7%)	​
ypN1	32 (27.4%)	26 (22.2%)	6 (5.1%)	​
ypN2	8 (6.8%)	6 (5.1%)	2 (1.7%)	​
ypN3	2 (1.7%)	1 (0.85%)	1 (0.85%)	​
Neoadjuvant target therapy	​	​	​	0.006
Trastuzumab	61 (52.1%)	57 (48.7%)	4 (3.4%)	​
Trastuzumab plus pertuzumab	56 (47.9%)	42 (35.9%)	14 (12.0%)	​
Neoadjuvant anthracycline	74 (63.2%)	62 (53.0%)	12 (10.2%)	0.744
Pathologic response	​	​	​	0.016
pCR	25 (21.4%)	25 (21.4%)	0	​
Non-pCR	92 (78.6%)	74 (63.2%)	18 (15.4%)	​
ctDNA	​	​	​	​
ctDNA (+) before NAT	79 (67.5%)	62 (53.0%)	17 (14.5%)	0.008
ctDNA (+) after NAT	32 (27.4%)	22 (18.8%)	10 (8.6%)	0.004

### Prognostic impact of ctDNA, pCR, and clinicopathologic factors

According to the results of the Katherine study, adjuvant T-DM1 is indicated for patients who do not achieve pCR after NAT (5). As T-DM1 has a strong impact on the survival outcome of patients without pCR, we divided our cohort into two groups: patients receiving adjuvant T-DM1 (*n* = 18) and those not receiving T-DM1 (*n* = 99). We analyzed the clinical impact of ctDNA on RFS in the non–T-DM1 cohort and explored the therapeutic effect of T-DM1 in terms of the clearance of ctDNA in patients treated with T-DM1.

**Figure 1.**
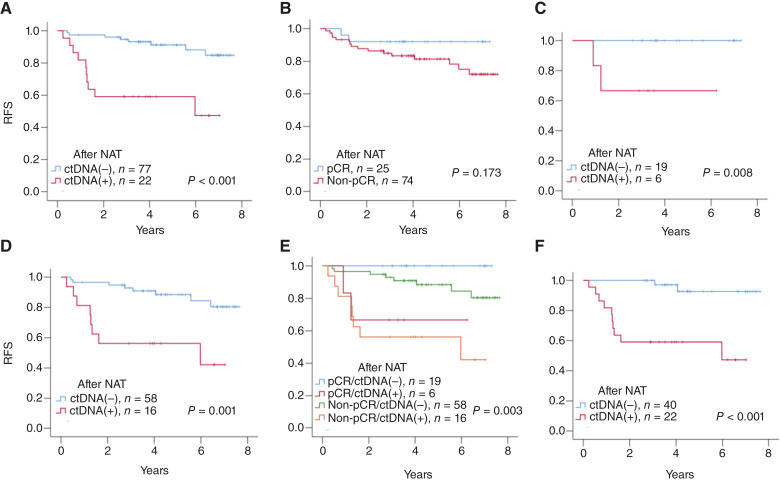
Illustration of RFS in the non–T-DM1 cohort (*n* = 99). RFS was estimated using the Kaplan–Meier method for the entire cohort according to (**A**) ctDNA after NAT and (**B**) pCR status. **C,** Among patients with pCR after NAT, ctDNA-positive results were associated with inferior survival (*P* = 0.008). **D,** ctDNA levels after NAT are associated with better survival, even in patients who did not achieve pCR (*P* = 0.001). **E,** When patients were stratified according to pCR and ctDNA status, HER2 patients with ctDNA-negative had a better RFS despite pCR status, whereas patients with ctDNA positivity had an inferior RFS. **F,** Among the 79 patients who were ctDNA-positive before NAT, those with ctDNA clearance had a significantly better RFS (*P* = 0.001).

In the patients without adjuvant T-DM1 (*n* = 99), the median follow-up after surgery was 4.02 years (range, 0.24–7.64 years). The 3-year RFS was 86.7% (95% CI, 80.0%–93.3%). Univariate analyses revealed that a residual tumor size greater than 5 cm (HR, 10.465; 95% CI, 1.324–82.690; *P* = 0.236), involved lymph nodes at surgery (HR, 6.08; 95% CI, 2.167–17.072; *P* = 0.001), and ctDNA positivity after NAT (HR, 6.045; 95% CI, 2.367–15.439; *P* < 0.001; Table 2; Fig. 1A) were poor prognostic factors for RFS. Non-pCR was associated with a trend toward inferior survival, but the association was not statistically significant (HR, 2.669; 95% CI, 0.613–11.619; *P* = 0.191; Table 2; Fig. 1B). Other factors did not affect RFS, such as ctDNA level before NAT, age, tumor size before NAT, lymph node status before NAT, and adjuvant trastuzumab or dual blockade (Table 2). RFS was similar between patients with and without *TP53*, *PIK3CA*, and other ctDNA mutations (Table 2).

**Table 2.** Univariate and multivariate analysis of clinical and pathologic parameters on the RFS in the 99 patients treated with adjuvant non–T-DM1.

​	Univariate analysis				Multivariate analysis			
	HR	95% CI		*P* value	HR	95% CI		*P* value
		Lower	Upper			Lower	Upper	
Age (>50 years vs. <50 years)	1.448	0.561	3.737	0.444	​	​	​	​
T classification (before NAT)	​	​	​	​	​	​	​	​
cT1	​	​	​	​	​	​	​	​
cT2	1.591	0.206	12.280	0.656	​	​	​	​
cT3-4	0.971	0.100	9.416	0.980	​	​	​	​
N classification (before NAT)	​	​	​	​	​	​	​	​
cN(−)	​	​	​	​	​	​	​	​
cN(+)	7.507	0.996	56.609	0.051	​	​	​	​
T classification (after NAT)	​	​	​	​	​	​	​	​
No tumor/ypTis	​	​	​	​	​	​	​	​
ypT1	5.439	0.680	43.513	0.110	11.661	0.602	225.688	0.104
yp≧T2	10.465	1.324	82.690	0.026	22.840	1.458	357.823	0.026
N classification (after NAT)	​	​	​	​	​	​	​	​
ypN(−)	​	​	​	​	​	​	​	​
ypN(+)	6.082	2.167	17.072	0.001	6.846	1.927	24.313	0.003
Response	​	​	​	​	​	​	​	​
pCR	​	​	​	​	​	​	​	​
Non-pCR	2.669	0.613	11.619	0.191	10.387	0.992	108.801	0.051
Gene mutation	​	​	​	​	​	​	​	​
*TP53*^a^	1.855	0.719	4.791	0.202	​	​	​	​
*PIK3CA*^a^	1.742	0.504	6.023	0.381	​	​	​	​
ctDNA	​	​	​	​	​	​	​	​
(+) before NAT^b^	1.376	0.513	3.688	0.526	​	​	​	​
(+) after NAT^b^	6.045	2.367	15.439	<0.001	5.505	1.950	15.540	0.001
Adjuvant regimen	​	​	​	​	​	​	​	​
Trastuzumab	​	​	​	​	​	​	​	​
Dual blockade	0.541	0.155	1.884	0.334	0.538	0.143	2.027	0.360

a Compared with no mutation.

b Compared with ctDNA(−).

A multivariate Cox regression model confirmed that ctDNA positivity after NAT (HR, 5.505; 95% CI, 1.950–15.540; *P* = 0.001) was an independent prognostic factor for predicting recurrence after incorporating residual tumor size, lymph node status at surgery, pCR status, adjuvant anti-HER2 regimen, and ctDNA positivity after NAT. Residual tumor size > 5 cm and positive lymph nodes after NAT were also significantly associated with recurrence (Table 2). Patients with a non-pCR status had a borderline significant risk of recurrence (HR, 10.387; 95% CI, 0.992–108.801; *P* = 0.051).

We analyzed the relationship between pCR and ctDNA positivity after NAT in the non–T-DM1 cohort. Consistent with our previous studies (15), patients who achieved pCR but were ctDNA-positive after NAT had a higher chance of recurrence than those who achieved pCR and were ctDNA-negative (log-rank test, *P* = 0.008; Fig. 1C). In contrast, patients who did not achieve pCR and were ctDNA-negative after NAT had better survival rates than those with ctDNA-positive results (log-rank test, *P* < 0.001; Fig. 1D). Comparing the four subgroups stratified by pCR and ctDNA (Fig. 1E), we found that there was no significant difference in RFS in patients who achieved pCR and ctDNA(−) and patients with non-pCR and ctDNA(−) (*P* = 0.108). In contrast, there was no difference in RFS between patients with pCR/ctDNA(+) and non-pCR/ctDNA(+) status (*P* = 0.662). This result suggests that ctDNA status plays an important role in RFS.

### ctDNA clearance by NAT was associated with a favorable outcome

Among the 99 patients, there were 62 patients harboring ctDNA positivity before NAT. We then analyzed the impact of ctDNA clearance after NAT on their prognosis. ctDNA clearance was defined as ctDNA positivity before NAT, and ctDNA negativity after NAT. Forty of the 62 patients had ctDNA clearance. Clearance of ctDNA was associated with significantly better RFS than that without clearance (*P* = 0.001, Fig. 1F).

### Adjuvant TDM-1 increased the ctDNA clearance rate and revert the poor prognosis of ctDNA positivity after NAT

In the overall cohort (*n* = 117), 32 patients had ctDNA-positive results after NAT, and 20 had at least one follow-up ctDNA measurement. Eight of the 20 patients received adjuvant T-DM1, and their ctDNA became negative during the follow-up period (ctDNA clearance, T-DM1 8/8 vs. non–T-DM1 7/12, *P* = 0.035).

**Figure 2.**
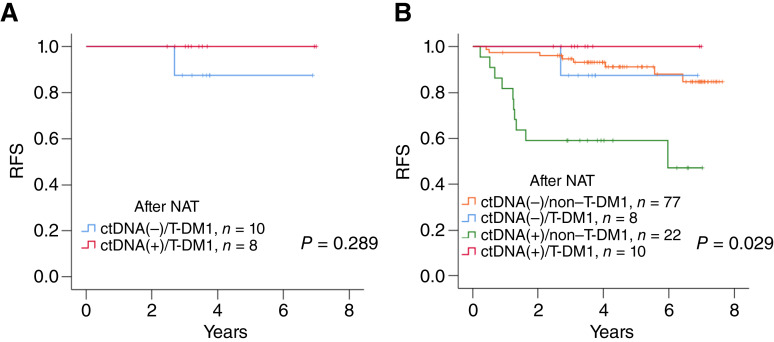
RFS in the patients treated with adjuvant T-DM1 (*n* = 18). **A,** RFS was estimated using the Kaplan–Meier method according to the ctDNA status after NAT. **B,** Stratifying patients by T-DM1/non–T-DM1 and ctDNA status, patients with ctDNA-positive/non–T-DM1 had significantly inferior RFS compared with the other three patient groups (*P* = 0.029).

Eighteen patients were treated with adjuvant T-DM1, and all of them had non-pCR after NAT. Ten patients were ctDNA-positive after NAT, whereas eight patients were ctDNA-negative after NAT. There was no difference in RFS between the two patient groups, suggesting that the poor risk of ctDNA diminished after T-DM1 therapy (Fig. 2A).

We compared the RFS of patients with and without T-DM1 therapy by stratifying them into four groups: ctDNA-negative/non–T-DM1 (*N* = 77), ctDNA-negative/T-DM1 (*N* = 8), ctDNA-positive/non–T-DM1 (*N* = 22), and ctDNA-positive/T-DM1 (*N* = 10; Fig. 2B). The 3-year RFS rates of patients with ctDNA-positive/non–T-DM1, ctDNA-positive/T-DM1, ctDNA-negative/non–T-DM1, and ctDNA-negative/T-DM1 were 59.1% (95% CI, 38.5%–79.7%), no recurrence (median follow-up 3.3 years), 93.2% (95% CI, 87.3%–99.1%), and 87.5% (95% CI, 64.6%–100.0%), respectively. The statistic result revealed that only patients with ctDNA-positive/non–T-DM1 had a significantly inferior RFS compared with that of the other three groups (log-rank test, *P* = 0.029). The 3-year RFS rates of the patients with ctDNA-positive/T-DM1, ctDNA-negative/T-DM1, and ctDNA-negative/non–T-DM1 were similar. These results suggest that adjuvant T-DM1 could reverse the poor prognostic outcome of ctDNA-positive patients after NAT.

### The ctDNA mutation and its dynamic changes associated with treatment response

**Figure 3.**
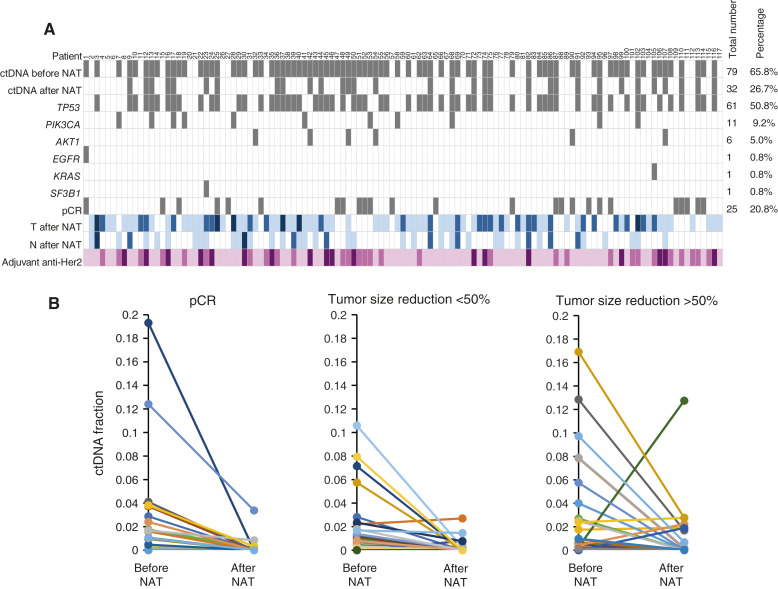
Illustration of the mutation profiles and the correlation with the clinical characteristics. **A,** Each column represents a patient. ctDNA positivity, gene mutations, and pCR are shown in gray. Tumor size after NAT was categorized by different colors: no tumor/Tis, white color, T1 pale blue; T2, blue; and T3/T4, oriental blue. Lymph node classification after NAT was marked as follows: no lymph nodes with white color, N1 with pale blue, N2 with blue, and N3 with oriental blue. For anti-HER2 adjuvant therapy, pink represents trastuzumab therapy, orchid represents trastuzumab plus pertuzumab, and dark azalea represents the T-DM1 treatment. **B,** Changes of the ctDNA fractions in between time points of the before and after NAT, according to the patients with pCR and residual cancer sizes <50% and >50%.

The spectrum of ctDNA mutations, along with their clinical and pathologic characteristics, is shown in Fig. 3A and Supplementary Table S1. Among the genes analyzed using the Oncomine Breast cfDNA Assay, 61 patients had *TP53* mutations, 11 had *PIK3CA* mutations, and nine had mutations in *AKT1*, *EGFR*, *KRAS*, and *SF3B1*. Five patients had somatic *TP53* and *PIK3CA*.

#### Association between ctDNA and tumor response after NAT

Among the 79 patients positive for ctDNA before NAT, the median allelic frequency of ctDNA mutations was 1.10% (range, 0.11%–19.3%). The median allelic frequency of the 32 patients who tested positive for ctDNA after NAT was 0.32% (range, 0.02%–12.7%). Tumor response was classified as pCR, tumor reduction >50%, or <50% decrease from the baseline longest dimension of the primary breast tumor. A positive correlation was observed between tumor reduction and ctDNA clearance. The proportions of ctDNA clearance were 75.0%, 74.2%, and 40.0% in patients who achieved pCR and tumor reductions of >50%, and <50%, respectively (*P* = 0.023, Fig. 3B).

#### Illustration of ctDNA dynamics during the serial follow-up after NAT

**Figure 4.**
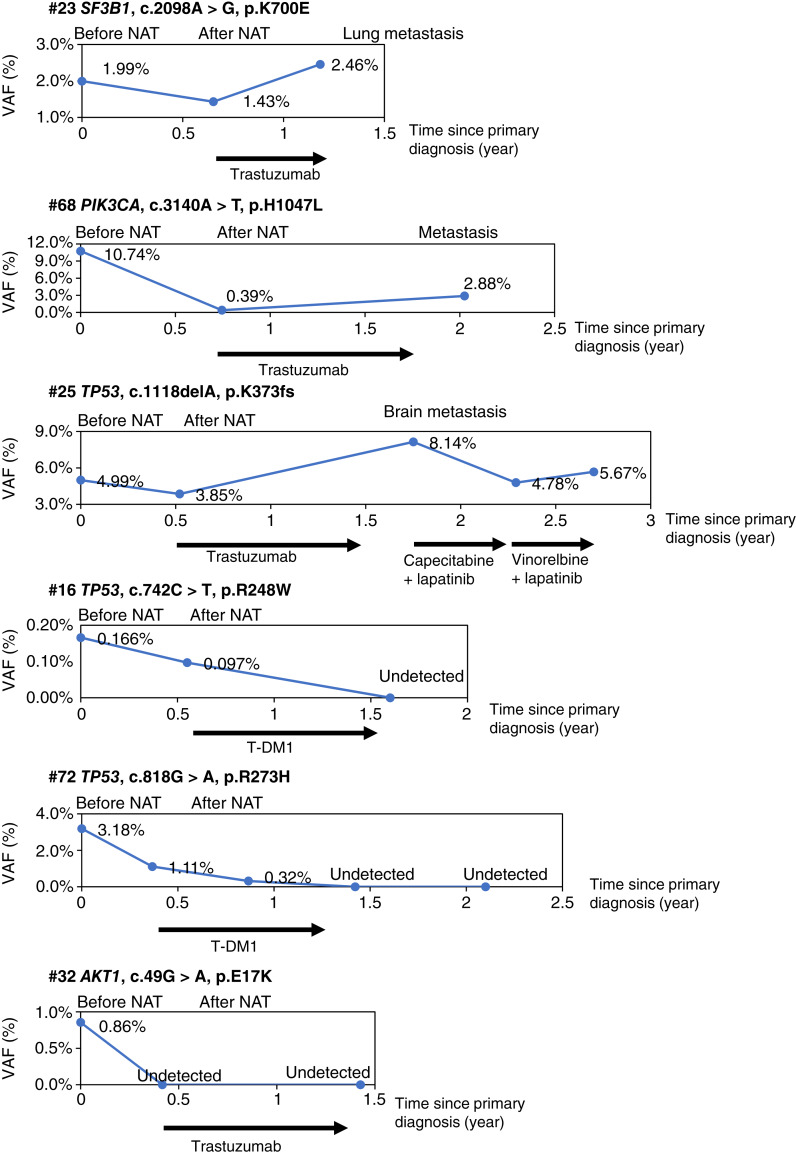
The dynamic changes of the ctDNA level. For each of the six patients with ctDNA detected in the plasma, longitudinal plots depicted the VAFs over time. VAF, estimated variant allele frequency.

Dynamic changes in allelic frequency for the targeted genetic variants are shown in Fig. 4. All three patients (patients #23, #68, and #25) who experienced recurrence showed persistently positive ctDNA results during follow-up. The allelic frequency of genetic variants decreased after NAT and subsequently increased at the time of metastasis. Patient #25 received trastuzumab as adjuvant therapy, and her TP53 mutation rate was elevated to 8.14% at the time of metastasis and decreased after salvage treatment with capecitabine and lapatinib. However, the allelic frequency increased with the clinical failure of salvage treatment. Patient #25 died because of progression of brain metastasis. Patients #16 and #72 showed positive ctDNA results after the NAT. Both patients received T-DM1, and their ctDNA positivity switched from detectable to undetectable levels and remained disease-free. Patient #32 had estrogen receptor–positive/HER2+ breast cancer, and *AKT1* (c.49G>A, p. E17K) mutations were detected in the ctDNA before NAT. This patient had residual invasive breast cancer and undetectable ctDNA levels after NAT. She received adjuvant trastuzumab after NAT and remained disease-free (disease-free for 3.1 years).

## Discussion

Our study demonstrated that the detection of ctDNA after NAT could predict recurrence of the HER2+ EBC. The prognostic value of ctDNA is more significant than that of pathologic assessment, including tumor size before NAT or at surgery, lymph node status, and even pCR. We also investigated the association between ctDNA levels, different adjuvant anti-HER2 regimens, and patient survival. The results showed that patients with ctDNA-positive results after NAT benefited more from adjuvant T-DM1 therapy, whereas patients with ctDNA-negative results had similar RFS regardless of whether they received adjuvant T-DM1 or non–T-DM1 therapy. Among patients who were ctDNA-positive and treated with T-DM1, ctDNA eventually became negative in the subsequent tests. To the best of our knowledge, this is the first study on ctDNA in HER2+ EBC with a significant sample size that demonstrated its impact on the selection of adjuvant therapy.

In EBC, ctDNA is released from primary breast tumors and from micrometastatic or circulating tumor cells (23). The presence of ctDNA indicates the presence of tumor cells either in the peripheral sites or in the primary residual tumor in patients after NAT (17). In this study, two patients who achieved pCR of the primary tumor but remained ctDNA-positive after NAT developed metastases during follow-up. All patients underwent a CT scan before NAT to exclude overt clinical metastasis. This observation suggests that ctDNA originates from micrometastatic or circulating tumor cells and aligns with previous reports, indicating that the presence of ctDNA may reflect significant minimal residual disease (MRD) and lead to recurrence (15, 17, 24–26). One possible concern is whether the PET scan might be more sensitive in detecting micrometastases in these two patients before NAT, so that the two patients had stage IV disease at the initial diagnosis. Another issue is that our pCR rate (21.4%) was relatively lower than that of the NAT trial for Her2 EBC (27). This may be due to the relatively less intense NAT regimen used in our cohort. Only 56 (47.9%) patients received dual blockade and 61 (52.1%) patients received trastuzumab. Seventy-four (63%) patients were treated with neoadjuvant epirubicin or doxorubicin–cyclophosphamide, whereas the other 43 patients were not. The relatively less intense NAT may also cause less clearance of ctDNA, which may explain why six patients achieved pCR but ctDNA(+) after NAT.

By contrast, ctDNA clearance after NAT is associated with a low recurrence rate. In our study, patients with ctDNA negativity after NAT had favorable survival rates despite having residual primary breast tumors, consistent with the findings of previous studies (10, 15). Among the 20 patients who were ctDNA-positive after NAT and underwent serial ctDNA testing, 15 tested negative and remained disease-free. In addition to breast cancer, patients who are ctDNA-negative after curative treatment have demonstrated better prognoses for early-stage colon, esophageal, and lung cancers (28–32). Evidence from these studies, along with the present data, confirms the prognostic significance of ctDNA in predicting the risk of recurrence.

Among patients who were ctDNA-positive after NAT, adjuvant T-DM1 therapy significantly reduced the risk of recurrence compared with adjuvant non–T-DM1 therapy. This suggests that the poor prognosis associated with ctDNA-positive results could be overcome by adjuvant T-DM1 and that ctDNA-positive status could serve as a biomarker to guide adjuvant therapy in HER2+ EBC.

Our study demonstrated similar survival rates between ctDNA-negative patients treated with adjuvant T-DM1 and non–T-DM1 despite their pCR status. Among the 92 patients without a pCR in our cohort, 66 were ctDNA-negative (71.7%) after NAT. In the I-SPY2 study, 86% of non-pCR patients who were ctDNA-negative after NAT had excellent survival, similar to those who achieved pCR (10). This observation suggests that a significant proportion of patients without pCR have a low risk of recurrence if their ctDNA is negative after NAT, and it is worth prospectively investigating the long-term survival of non-pCR/ctDNA-negative patients.

With serial samples from some patients, we were able to demonstrate that adjuvant T-DM1 helped to clear ctDNA and improve survival, whereas patients who remained ctDNA-positive had a poor prognosis. One concern in the clinical monitoring of MRD using ctDNA is the possibility of false-positive or false-negative results. The most commonly discussed issue is the false positivity of ctDNA caused by the presence of clonal hematopoiesis of indeterminate potential (CHIP), which is the accumulation of plasma DNA mutations in both healthy individuals and patients with cancer (33–35). Six patients with pCR were ctDNA(+) after NAT, and five of them had a TP53 mutation in the ctDNA. A false-positive result should be considered because TP53 mutation might originate from CHIP. The ideal approach is to sequence tumor DNA in parallel; however, biopsy samples often have limited residual tumors after IHC staining and FISH examination. We previously established a pipeline for ctDNA analysis that helps reduce the false-positive rate of ctDNA detection (15). Furthermore, we observed that the allelic frequency of ctDNA increased at the time of recurrence and was correlated with the therapeutic response (Fig. 4). CHIP variants do not usually correlate with chemotherapy response (36). The maximum estimated incidence of detectable CHIP in patients with breast cancer is 25%, and TP53 mutations are present in 5% to 10% of patients with CHIP (37). Therefore, only 1.25% to 2.5% of our patients were expected to have potential TP53 CHIP mutations (37). We believe that TP53 CHIP had a limited effect in our study and did not affect the results.

In contrast, a significant proportion of patients were ctDNA-negative before NAT, and some patients without pCR were ctDNA-negative after NAT. First, except the common mutations such as *TP53*, *PIK3CA*, and *AKT1* mutations, a small proportion of patients with HER2 breast cancer did not carry any detectable mutation (38). Second, the Oncomine Breast cfDNA panel did not contain all the mutated genes reported in patients with HER2 breast cancer. The detection rate of ctDNA before NAT was 67.5% in our cohort. Thus, some patients may have experienced false-negative ctDNA results owing to the lack of detectable mutations or limited gene coverage of the sequencing panel. Caution should be exercised when considering the de-escalation of treatment using ctDNA. However, for ctDNA-positive samples before NAT, we found that ctDNA clearance was associated with a favorable RFS (10, 15). This indicates that ctDNA clearance is a valuable biomarker to guide therapy.

Since the Katherine trial showed that T-DM1 improved the survival of patients who did not achieve pCR after NATHER2, NAT has become the standard of care for the majority of patients with HER2+ EBC, and pCR is the primary factor in determining the need for adjuvant T-DM1 therapy (6). However, pCR results can be affected by false-negative findings during pathologic examination or improper localization of residual tumors during surgery, resulting in patients not receiving T-DM1 (11). Additionally, pathologic examination only assesses the therapeutic response of the local breast tumor, which might underestimate systemic micrometastasis in patients who achieve pCR. Our study demonstrated that ctDNA is an independent prognostic factor and may serve as an additional marker for guiding adjuvant T-DM1 treatment. Further large-scale collection of real-world data is necessary to confirm our findings. Future neoadjuvant/adjuvant trials investigating novel agents should consider incorporating ctDNA as a marker of MRD in addition to non-pCR (22).

## Supplementary Material

Supplementary Table 1 ctDNA results, adjuvant anti-Her2 regimens and recurrence outcomes for all patients

Supplementary Figure 1 Supplementary Figure 1. Distribution of the entire 117 patients based on pCR and ctDNA status.

## Data Availability

Information on individual ctDNA mutations, disease status, and survival time is listed in Supplementary Table S1. The sequencing data (BAM files) of the ctDNA in this study are submitted to NCBI BioProject number SUB15705997. Other information in this study is available upon the request to the corresponding author.
