# Supplementary material for: ctDNA Detected after Neoadjuvant Therapy for HER2-Positive Breast Cancer Is Associated with Inferior Outcomes and May Inform Adjuvant Therapy
Source: Cancer Res Commun. 2026 Jan 14;6(1):105–14. doi: 10.1158/2767-9764.CRC-24-0234 (PMC12802552; doi:10.1158/2767-9764.CRC-24-0234)
Supplement: Supplementary Table 1 — ctDNA results, adjuvant anti-Her2 regimens and recurrence outcomes for all patients [file crc-24-0234_supplementary_table_1_suppst1.docx]

Supplementary Table 1. ctDNA results, adjuvant anti-Her2 regimens and recurrence outcomes for all patients

| patient | gene | cDNA | amino acid | ctDNA_before_NAT | ctDNA_after_NAT | adjuvant Her2 regimen | recurrence | Time to recurrence or last follow-up (years) |
| --- | --- | --- | --- | --- | --- | --- | --- | --- |
| #1 | *EGFR* | c.2565delT | p.D855fs | positive | negative | non-T-DM1 | No | 3 |
| #2 |  |  |  | negative | negative | non-T-DM1 | Yes | 2.06 |
| #3 | *TP53* | c.1114A>T | p.K372* | positive | negative | non-T-DM1 | Yes | 3.08 |
| #4 |  |  |  | negative | negative | non-T-DM1 | No | 6.93 |
| #5 |  |  |  | negative | negative | non-T-DM1 | No | 6.52 |
| #6 |  |  |  | negative | negative | non-T-DM1 | No | 7.2 |
| #7 | *PIK3CA* | c.3140A>G | p.H1047R | positive | negative | non-T-DM1 | No | 4.5 |
| #8 |  |  |  | negative | negative | T-DM1 | Yes | 2.69 |
| #9 | *TP53* | c.736A>G | p.M246V | positive | positive | non-T-DM1 | Yes | 5.97 |
| #10 | *TP53* | c.537delA | p.H179fs | positive | negative | non-T-DM1 | No | 7.42 |
| #11 |  |  |  | negative | negative | non-T-DM1 | No | 6.72 |
| #12 | *TP53* | c.992_993insT | p.Q331fs | positive | positive | T-DM1 | No | 3.2 |
| #13 | *TP53* | c.524G>A | p.R175H | positive | positive | non-T-DM1 | No | 7.03 |
|  | *PIK3CA* | c.1637A>G | p.Q546R |  |  |  |  |  |
| #14 | *TP53* | c.524G>A | p.R175H | positive | negative | non-T-DM1 | No | 6.7 |
| #15 |  |  |  | negative | negative | non-T-DM1 | No | 5.65 |
| #16 | *TP53* | c.742C>T | p.R248W | positive | positive | T-DM1 | No | 2.47 |
| #17 | *TP53* | c.993+2T>C |  | positive | positive | non-T-DM1 | No | 6.59 |
|  | *PIK3CA* | c.1633G>A | p.E545K |  |  |  |  |  |
| #18 | *TP53* | c.1009C>T | p.R337C | positive | negative | non-T-DM1 | No | 4.51 |
| #19 | *PIK3CA* | c.1634A>G | p.E545G | positive | negative | non-T-DM1 | No | 3.99 |
| #20 |  |  |  | negative | negative | non-T-DM1 | No | 6.9 |
| #21 |  |  |  | negative | negative | non-T-DM1 | No | 7.2 |
| #22 | *TP53* | c.424C>A | p.P142T | positive | negative | T-DM1 | No | 2.93 |
| #23 | *SF3B1* | c.2098A>G | p.K700E | positive | positive | non-T-DM1 | Yes | 0.53 |
| #24 | *TP53* | c.254delC | p.P85fs | positive | negative | T-DM1 | No | 3.22 |
| #25 | *TP53* | c.1118delA | p.K373fs | positive | positive | non-T-DM1 | Yes | 1.23 |
| #26 |  |  |  | negative | negative | non-T-DM1 | No | 5.34 |
| #27 |  |  |  | negative | negative | non-T-DM1 | No | 6.8 |
| #28 | *PIK3CA* | c.1633G>A | p.E545K | positive | negative | non-T-DM1 | No | 7.64 |
| #29 | *TP53* | c.254delC | p.P85fs | positive | negative | non-T-DM1 | No | 4.58 |
| #30 | *TP53* | p.V97F | c.289G>T | positive | negative | T-DM1 | No | 6.89 |
| #31 |  |  |  | negative | negative | non-T-DM1 | No | 6.84 |
| #32 | *AKT1* | c.49G>A | p.E17K | positive | negative | non-T-DM1 | No | 3.13 |
| #33 | *TP53* | c.310C>T | p.Q104* | positive | negative | non-T-DM1 | No | 7.3 |
| #34 |  |  |  | negative | negative | non-T-DM1 | No | 4.28 |
| #35 | *TP53* | c.548C>A | p.S183* | positive | negative | T-DM1 | No | 3.66 |
| #36 | *TP53* | c.548C>G | p.S183* | positive | positive | non-T-DM1 | No | 4.29 |
| #37 | *TP53* | p.Y234C | c.701A>G | positive | positive | non-T-DM1 | Yes | 0.24 |
| #38 | *TP53* | c.992_993insT | p.Q331fs | positive | negative | non-T-DM1 | No | 7.11 |
| #39 | *TP53* | c.560-1G>A |  | positive | negative | non-T-DM1 | Yes | 4.06 |
| #40 | *TP53* | c.254delC | p.P85fs | positive | negative | non-T-DM1 | No | 4.04 |
| #41 | *PIK3CA* | c.1637A>G | p.Q546R | positive | negative | non-T-DM1 | No | 2.72 |
| #42 | *AKT1* | c.49G>A | p.E17K | positive | positive | T-DM1 | No | 3.43 |
| #43 | *TP53* | c.254delC | p.P85fs | positive | negative | non-T-DM1 | No | 3.73 |
| #44 | *TP53* | c.992_993insT | p.Q331fs | positive | positive | non-T-DM1 | Yes | 1.62 |
| #45 | *TP53* | c.313G>A | p.G105S | positive | negative | T-DM1 | No | 3.76 |
| #46 | *TP53* | c.535_537delCAT | p.H179del | positive | negative | T-DM1 | No | 3.75 |
| #47 |  |  |  | positive | negative | non-T-DM1 | No | 7 |
| #48 | *TP53* | c.524G>A | p.R175H | positive | positive | non-T-DM1 | No | 3.27 |
| #49 | *AKT1* | c.49G>A | p.E17K | positive | positive | non-T-DM1 | No | 3.93 |
| #50 | *TP53* | c.188C>A | p.A63D | positive | positive | T-DM1 | No | 3.11 |
| #51 | *TP53* | c.772G>T | p.E258* | positive | negative | non-T-DM1 | No | 5.17 |
| #52 | *TP53* | c.577C>T | p.H193Y | positive | negative | non-T-DM1 | No | 4.63 |
| #53 | *PIK3CA* | c.1633G>A | p.E545K | positive | negative | non-T-DM1 | No | 3.6 |
| #54 | *AKT1* | c.49G>A | p.E17K | positive | positive | non-T-DM1 | No | 2.91 |
| #55 | *TP53* | c.992_993insT | p.Q331fs | positive | negative | non-T-DM1 | No | 3.5 |
| #56 | *TP53* | c.1109A>G | p.K370R | positive | negative | non-T-DM1 | No | 6.66 |
| #57 |  |  |  | negative | negative | non-T-DM1 | No | 3.68 |
| #58 | *PIK3CA* | c.3140A>G | p.H1047R | positive | negative | non-T-DM1 | No | 4.47 |
| #59 |  |  |  | negative | negative | non-T-DM1 | No | 7.54 |
| #60 | *TP53* | c.104_105insC | p.L35fs | positive | negative | non-T-DM1 | No | 7.47 |
| #61 |  |  |  | negative | negative | non-T-DM1 | Yes | 0.51 |
| #62 | *TP53* | c.1101-1G>AGG |  | positive | negative | non-T-DM1 | No | 3.5 |
| #63 | *TP53* | c.844C>T | p.R282W | positive | negative | non-T-DM1 | No | 7.2 |
| #64 | *TP53* | c.470T>C | p.Val157Ala | positive | positive | non-T-DM1 | Yes | 1.25 |
| #65 |  |  |  | negative | negative | non-T-DM1 | No | 7.02 |
| #66 | *TP53* | c.734G>T | p.G245V | positive | negative | non-T-DM1 | No | 2.75 |
| #67 |  |  |  | negative | negative | non-T-DM1 | No | 5.2 |
| #68 | *TP53* | c.818G>A | p.R273H | positive | positive | non-T-DM1 | Yes | 1.28 |
|  | *PIK3CA* | c.3140A>T | p.H1047L |  |  |  |  |  |
| #69 | *TP53* | c.524G>A | p.R175H | positive | negative | non-T-DM1 | No | 2.93 |
| #70 |  |  |  | negative | negative | non-T-DM1 | Yes | 2.74 |
| #71 | *TP53* | c.524G>A | p.R175H | positive | negative | non-T-DM1 | No | 5.53 |
| #72 | *TP53* | c.818G>A | p.R273H | positive | positive | T-DM1 | No | 3.68 |
| #73 |  |  |  | negative | negative | non-T-DM1 | No | 6.95 |
| #74 | *TP53* | c.924delG | p.N310fs | positive | positive | T-DM1 | No | 6.94 |
| #75 | *TP53* | c.992_993insT | p.Q331fs | positive | positive | non-T-DM1 | No | 4.02 |
| #76 |  |  |  | negative | negative | non-T-DM1 | Yes | 5.57 |
| #77 |  |  |  | negative | negative | non-T-DM1 | No | 7.36 |
| #78 |  |  |  | negative | negative | non-T-DM1 | Yes | 6.42 |
| #79 | *TP53* | c.844C>T | p.R282W | positive | negative | non-T-DM1 | No | 7.1 |
| #80 |  |  |  | negative | negative | non-T-DM1 | Yes | 0.42 |
| #81 |  |  |  | negative | negative | non-T-DM1 | No | 7.07 |
| #82 | *TP53* | c.992_993insT | p.Q331fs | positive | positive | T-DM1 | No | 7 |
| #83 |  |  |  | negative | negative | non-T-DM1 | No | 7.46 |
| #84 | *TP53* | c.167_168delAA | p.E56fs | positive | negative | non-T-DM1 | No | 7.02 |
| #85 | *TP53* | c.904G>T | p.G302W | positive | negative | non-T-DM1 | No | 4.08 |
| #86 | *TP53* | c.517G>A | p.V173M | positive | positive | non-T-DM1 | Yes | 0.68 |
| #87 | *TP53* | c.163A>T | p.T55S | positive | positive | non-T-DM1 | No | 6.24 |
| #88 |  |  |  | negative | negative | non-T-DM1 | No | 6.97 |
| #89 |  |  |  | negative | negative | non-T-DM1 | No | 5.04 |
| #90 | *AKT1* | c.49G>A | p.E17K | positive | negative | non-T-DM1 | No | 3.63 |
| #91 | *TP53* | c.292C>G | p.P98A | positive | positive | non-T-DM1 | No | 6.56 |
| #92 |  |  |  | negative | negative | non-T-DM1 | No | 4.71 |
| #93 |  |  |  | negative | negative | non-T-DM1 | No | 3.97 |
| #94 | *TP53* | c.583_584insC | p.I195fs | positive | negative | non-T-DM1 | No | 4.15 |
| #95 | *TP53* | c.833C>G | p.P278R | positive | positive | non-T-DM1 | Yes | 0.9 |
|  | *PIK3CA* | c.3140A>G | p.H1047R |  |  |  |  |  |
| #96 |  |  |  | negative | negative | non-T-DM1 | No | 5.15 |
| #97 | *TP53* | c.535C>G | p.H179D | positive | negative | non-T-DM1 | No | 3.41 |
| #98 |  |  |  | positive | negative | non-T-DM1 | No | 3.81 |
| #99 |  |  |  | positive | positive | T-DM1 | No | 3.02 |
| #100 | *TP53* | c.277delC | p.L93fs | positive | negative | non-T-DM1 | No | 4.29 |
| #101 |  |  |  | negative | negative | non-T-DM1 | No | 4.3 |
| #102 | *TP53* | c.981T>A | p.Y327* | positive | positive | non-T-DM1 | Yes | 1.33 |
|  | *PIK3CA* | c.3140A>G | p.H1047R |  |  |  |  |  |
| #103 | *TP53* | c.1008G>C | p.E336D | positive | negative | non-T-DM1 | No | 2.71 |
| #104 |  |  |  | negative | negative | non-T-DM1 | No | 3.89 |
| #105 | *KRAS* | c.37G>A | p.G13S | positive | positive | non-T-DM1 | No | 3.82 |
| #106 | *TP53* | c.645T>A | p.S215R | positive | negative | T-DM1 | No | 3.55 |
| #107 | *AKT1* | c.49G>A | p.E17K | positive | positive | T-DM1 | No | 3.53 |
| #108 | *TP53* | c.992_993insT | p.Q331fs | positive | negative | non-T-DM1 | No | 3.53 |
| #109 |  |  |  | negative | negative | non-T-DM1 | No | 3.55 |
| #110 | *TP53* | c.274C>G | p.P92A | positive | positive | non-T-DM1 | No | 3.52 |
| #111 |  |  |  | negative | negative | non-T-DM1 | No | 3.03 |
| #112 |  |  |  | negative | negative | non-T-DM1 | No | 0.92 |
| #113 | *TP53* | c.171C>A | p.Asp57Glu | positive | positive | non-T-DM1 | No | 2.88 |
| #114 | *TP53* | c.1173C>G | p.L391(=) | positive | negative | non-T-DM1 | No | 2.6 |
| #115 |  |  |  | negative | negative | non-T-DM1 | No | 2.6 |
| #116 | *TP53* | c.274C>G | p.P92A | positive | positive | T-DM1 | No | 2.7 |
| #117 |  |  |  | negative | negative | non-T-DM1 | No | 2.65 |
