# Supplementary material for: ctDNA Detected after Neoadjuvant Therapy for HER2-Positive Breast Cancer Is Associated with Inferior Outcomes and May Inform Adjuvant Therapy
Source: Cancer Res Commun. 2026 Jan 14;6(1):105–14. doi: 10.1158/2767-9764.CRC-24-0234 (PMC12802552; doi:10.1158/2767-9764.CRC-24-0234)
Supplement: Supplementary Figure 1 — Distribution of the entire 117 patients based on pCR and ctDNA status. [file crc-24-0234_supplementary_figure_1_suppsf1.pptx]

## Slide 1
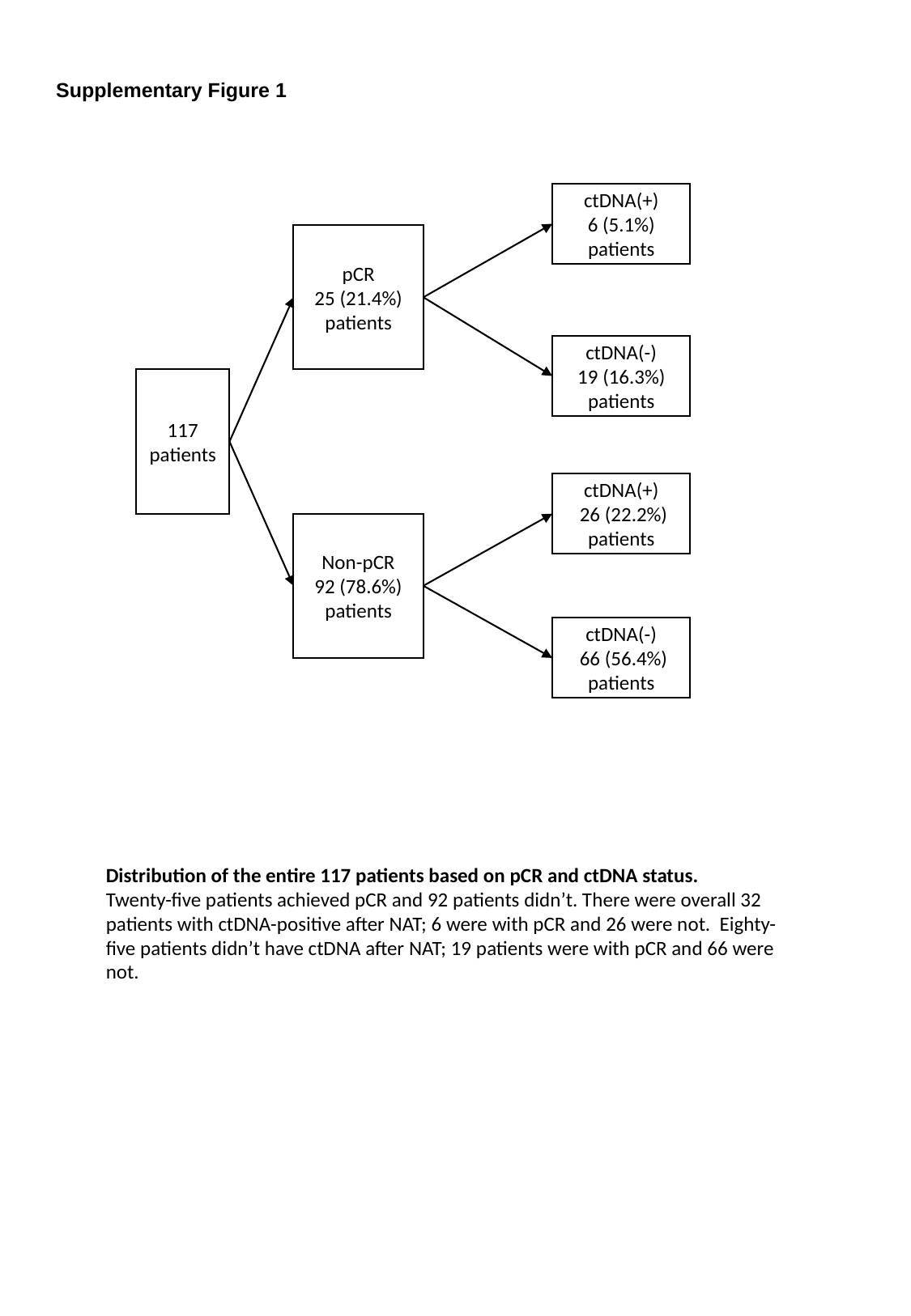

Supplementary Figure 1
ctDNA(+)
6 (5.1%) patients
pCR
25 (21.4%) patients
ctDNA(-)
19 (16.3%) patients
117 patients
ctDNA(+)
 26 (22.2%) patients
Non-pCR
92 (78.6%) patients
ctDNA(-)
 66 (56.4%) patients
Distribution of the entire 117 patients based on pCR and ctDNA status.
Twenty-five patients achieved pCR and 92 patients didn’t. There were overall 32 patients with ctDNA-positive after NAT; 6 were with pCR and 26 were not. Eighty-five patients didn’t have ctDNA after NAT; 19 patients were with pCR and 66 were not.

## Slide 2
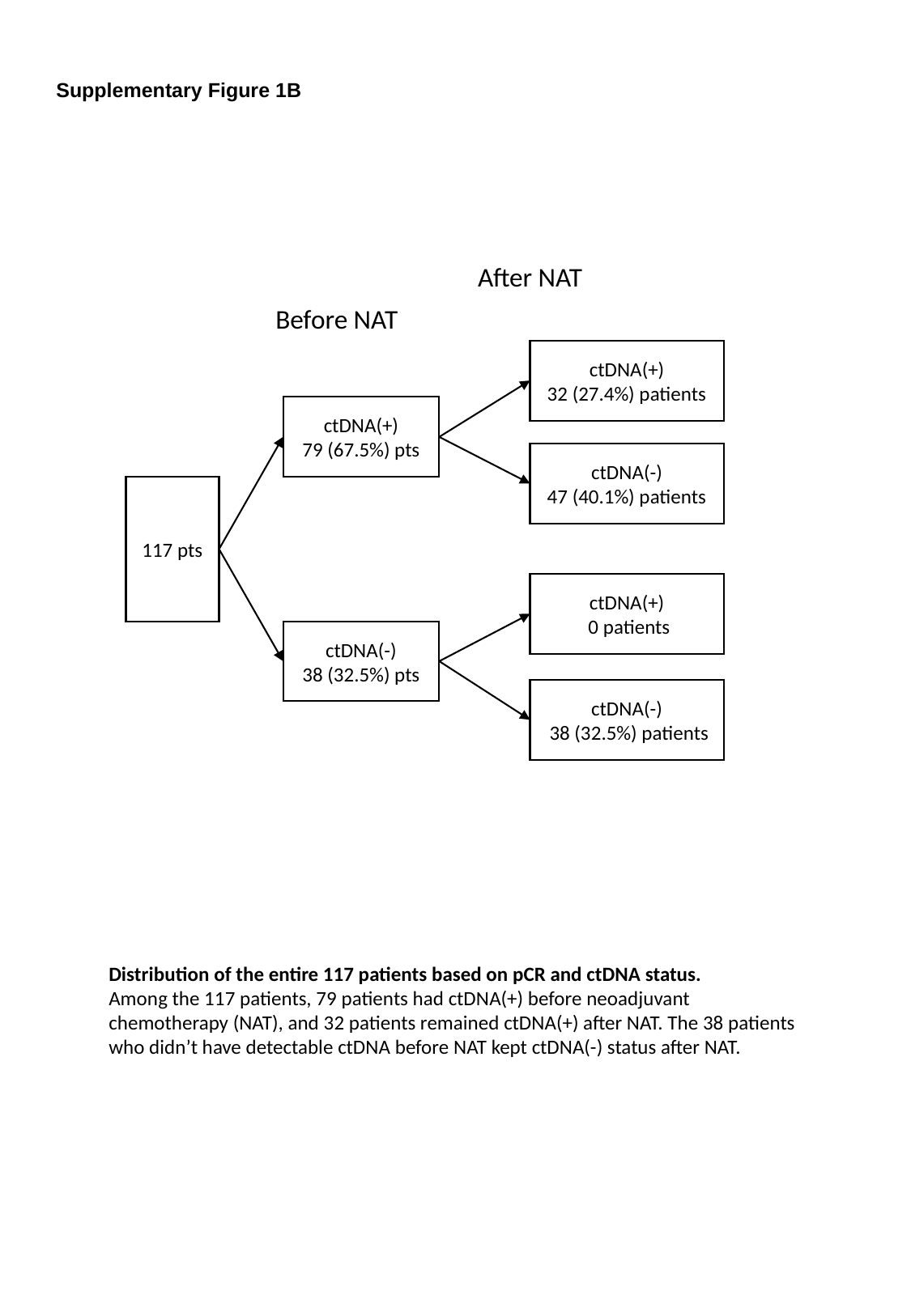

Supplementary Figure 1B
After NAT
Before NAT
ctDNA(+)
32 (27.4%) patients
ctDNA(+)
79 (67.5%) pts
ctDNA(-)
47 (40.1%) patients
117 pts
ctDNA(+)
 0 patients
ctDNA(-)
38 (32.5%) pts
ctDNA(-)
 38 (32.5%) patients
Distribution of the entire 117 patients based on pCR and ctDNA status.
Among the 117 patients, 79 patients had ctDNA(+) before neoadjuvant chemotherapy (NAT), and 32 patients remained ctDNA(+) after NAT. The 38 patients who didn’t have detectable ctDNA before NAT kept ctDNA(-) status after NAT.
